# Bone Response to Two Dental Implants with Different Sandblasted/Acid-Etched Implant Surfaces: A Histological and Histomorphometrical Study in Rabbits

**DOI:** 10.1155/2017/8724951

**Published:** 2017-12-27

**Authors:** Antonio Scarano, Adriano Piattelli, Alesandro Quaranta, Felice Lorusso

**Affiliations:** ^1^Department of Medical, Oral and Biotechnological Sciences and CeSI-MeT, University of Chieti-Pescara, Via dei Vestini 31, 66100 Chieti, Italy; ^2^Department of Medical, Oral and Biotechnological Sciences, University of Chieti-Pescara, Chieti, Italy; ^3^Division of Periodontics and Implantology, Oral Health Centre, Nedlands, WA, Australia

## Abstract

**Background:**

Scientific evidence in the field of implant dentistry of the past 20 years established that titanium rough surfaces have shown improved osseointegration rates. In a majority of dental implants, the surface microroughness was obtained by grit blasting and/or acid etching. The aim of the study was to evaluate* in vivo *two different highly hydrophilic surfaces at different experimental times.

**Methods:**

Calcium-modified (CA) and SLActive surfaces were evaluated and a total of 18 implants for each type of surface were positioned into the rabbit articular femoral knee-joint in a split model experiment, and they were evaluated histologically and histomorphometrically at 15, 30, and 60 days of healing.

**Results:**

Bone-implant contact (BIC) at the two-implant surfaces was significantly different in favor of the CA surface at 15 days (*p* = 0.027), while SLActive displayed not significantly higher values at 30 (*p* = 0.51) and 60 days (*p* = 0.061).

**Conclusion:**

Both implant surfaces show an intimate interaction with newly formed bone.

## 1. Introduction

The clinical success of titanium dental implants is based on a high percentage of bone/implant contact [1], and for this purpose, dental implants surfaces have been treated in order to trigger cellular actions and enhance the proper integration of the implant with the surrounding bone. Dental implants with microrough titanium surfaces have paved the way for further development of surface topographies to promote an enhanced peri-implant bone apposition during the early stages of bone formation [2].

Using histomorphometry to measure the percentage of bone-to-implant contact (BIC) is an established method to determine the extent of osseointegration and the rate of healing of dental implants. Experimental studies in animal models have shown that implants with roughened surfaces have a better early anchorage in bone tissue and a higher percentage of BIC than implants with smooth surfaces [3]. These results have also been demonstrated in human studies [4].

The implant surface roughness has been considered as one of the most relevant aspects in establishing a clinically reliable bone attachment [5]. Different methods can be used to roughen a surface, such as electrochemical deposition, sandblasting with abrasives, acid etching, or combinations of such treatments. The increased surface area of such designs provides a greater potential for cell attachment, and tissue ingrowth into the implant surface would be expected to stabilize the device mechanically. Fibroblasts shunned such roughened surfaces and accumulated on the smooth portions of the tissue culture dish [6, 7]. In contrast, macrophages preferred the rough surfaces to the smooth ones, a behavior that has been described as “rugophilia” [8]. The surfaces provided for cell attachment can directly affect cell shape and cell function. Cells grown on grooved substrata are more round than cells grown on flat, smooth substrata [9]. A number of cellular properties, including growth [10], secretion of proteinases [9], and gene expression [11], are affected by cell shape. The surface texture on an implant has the potential of specifically selecting a certain population of cells and altering their functions [12].

Surface blasting and acid etching can increase the rate and amount of bone formation on the implant surface [13]. Wennerberg et al. have reported a significantly greater BIC at 2 and 4 weeks of healing in implants with a chemically modified sandblasted/acid-etched (SLActive) as compared with the standard SLA surface [14]. Many comparative studies have been conducted between smooth, sandblasted, and SLA implant surfaces showing an increase of BIC around SLA implant surface [15, 16]. In 2011, Wennerberg et al., summarizing the current knowledge about the SLA surface, reported the presence of 15 in vitro studies, 14 in vivo animal studies, 3 experimental studies in humans, and 16 clinical trials [17].

The purpose of this study was to compare in vivo the BIC between two different surfaces using a split implant design.

## 2. Materials and Methods

### 2.1. Surface Preparation by Manufacturer

The two-implant surface used in this study was prepared by manufactures. The chemically activated calcium-modified surface (CA) (Osstem Implant Co., Ltd., Busan, Korea) was prepared by a controlled process for the protection of carbon adsorption after surface treatment (test group). The CA surface was prepared by sandblasting with 250~500 *μ*m Al_2_O_3_ grit and acid etching in hydrochloric and sulfuric acid according to the proprietary process. The implants after sandblasting and acid etching were rinsed under protective environment for preventing carbon adsorption on Ti surface and then stored in CaCl_2_ solution. The process resulted in a more active hydrophilic surface, with higher surface energy and less hydrocarbon contamination from atmospheric environment. As a control group, implants with an SLActive surface are produced using coarse grit blasting with 0.25–0.5 mm corundum followed by a subsequent acid conditioning with sulfuric and hydrochloric acids; the implants were then rinsed under nitrogen protection to prevent exposure to air and are then stored in a sealed glass tube containing isotonic saline solution (Straumann, Basel, Switzerland) [18].

### 2.2. SEM and Electron Spectroscopy Evaluation

Ten implants SLActive and 10 CA implants were used for the evaluation of the surface topography by scanning electron microscopy (SEM, JSM-6480LV; Jeol, Tokyo, Japan) that is characterized by a solid state backscattered detector operated in 20 kV accelerating voltage. Instead, the chemical composition of surfaces was evaluated by electron spectroscopy (AES, PHI 700; ULVAC-PHI Inc., Kanagawa, Japan) employing a 10 kV/10 nA electron beam energy to characterize the near surface (0.5–3.0 *µ*) elemental composition. All dental implants were taken from their original package directly from the supplier. Each implant was attached on an aluminum stub with sticky conductive carbon tape. The surface of each implant was examined with a field emission environmental scanning electron microscope. Pictures were taken in both secondary and backscattered electrons.

### 2.3. In Vivo Experiment

Eighteen mature New Zealand white male rabbits, weighing about 2.5 Kg, were used in this study. The study was approved by the Ethical Committee of the University of Chieti-Pescara, Chieti, Italy. A total of 36 implants with two different surfaces, CA Implants (Osstem) and SLActive (Straumann), were used. Eighteen implants of each different surface were used. The implants were inserted, in a random fashion, into the articular femoral knee-joint. All animals before the surgical procedure were anesthetized with intramuscular injections of fluanizone (0,7 mg/kg b.wt.) and diazepam (1.5 mg/kg b.wt.), and local anaesthesia was given using 1 ml of 2% lidocaine/adrenaline solution. A skin incision with a periosteal flap was used to expose the articular surface. The preparation of the bone defect was done with burs under generous saline irrigation. Each rabbit received two implants, one in each knee joint. During the course of the experiment 2 rabbits died; these rabbits were substituted. The animals were killed after 15, 30, and 60 days, with an intravenous injection of Tanax. A total of 36 implants were retrieved.

The implants and surrounding tissues were stored immediately in 10% buffered formalin and processed to obtain thin ground sections. The specimens were processed using the Precise 1 Automated System (Assing, Rome, Italy) [19]. The specimens were dehydrated in a graded series of ethanol rinses and embedded in a glycol methacrylate resin (Technovit 7200 VLC, Kulzer, Wehrheim, Germany). After polymerization, the specimens were sectioned, along the longitudinal axis of the implants, with a high-precision diamond disc at about 150 *μ*m and ground down to about 30 *μ*m with a specially designed grinding machine (Assing, Rome, Italy). Three slides were obtained for each implant. These slides were stained with acid fuchsin and toluidine blue and examined with transmitted light under a Leitz Laborlux microscope (Leitz, Wetzlar, Germany).

*Bone-implant contact* was carried out using a light microscope (Laborlux S, Leitz, Wetzlar, Germany) connected to a high resolution video camera (3CCD, JVC KY-F55B, JVC®, Yokohama, Japan) and interfaced to a monitor and PC (Intel Pentium III 1200 MMX, Intel®, Santa Clara, CA, USA). This optical system was associated with a digitizing pad (Matrix Vision GmbH, Oppenweiler, Germany) and a histometry software package with image capturing capabilities (Image-Pro Plus 4.5, Media Cybernetics Inc., Immagini & Computer Snc Milano, Italy). A total of 18 implants for each type of surface were analysed.

### 2.4. Statistical Evaluation

To evaluate the differences between the bone-to-implant contact (BIC) percentages between the groups, Student's *t*-test was used. Significance was set at *p* < 0.05.

## 3. Results

### 3.1. SEM and X-Ray Spectroscopy Evaluation

#### 3.1.1. CA Surface

**Figure 1:**
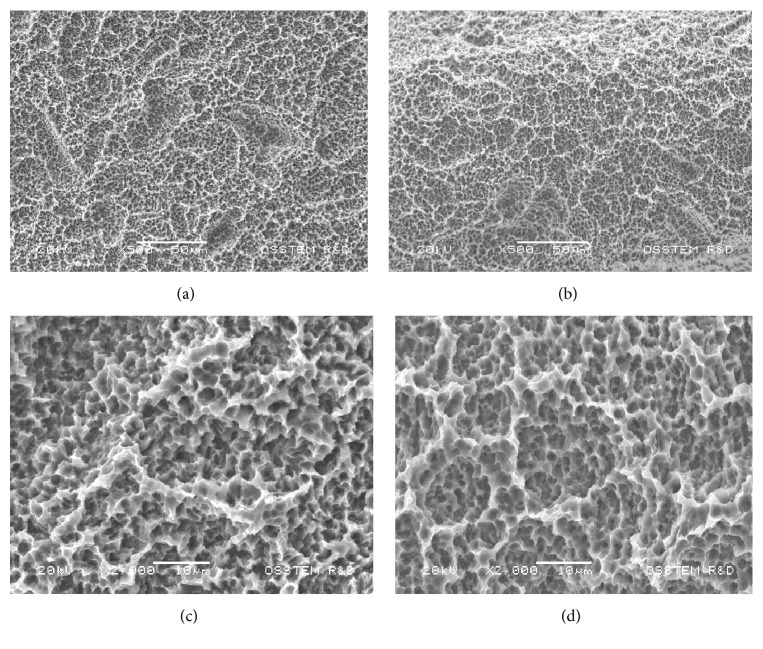
SEM analysis of CA (a and b) and SLActive (c and d) surfaces at lower (500x) and higher (2000x) magnifications. Both surfaces presented an irregular surface, with rounded grooves and craterlike micropores.

Ten micrographs of this surface were examined. CA surface roughness (Sa) was 2.69 ± 0.31 *μ*m, with irregularly rounded shape domains (Figure 1). At high magnifications, an oxide film formed during the acidic treatment after sandblasting was observed. No microcracks on the surface were observed. X-ray spectroscopy (XPS) analysis indicated increased Ca concentrations on the surface (2.03%) (Figure 3).

**Figure 2:**
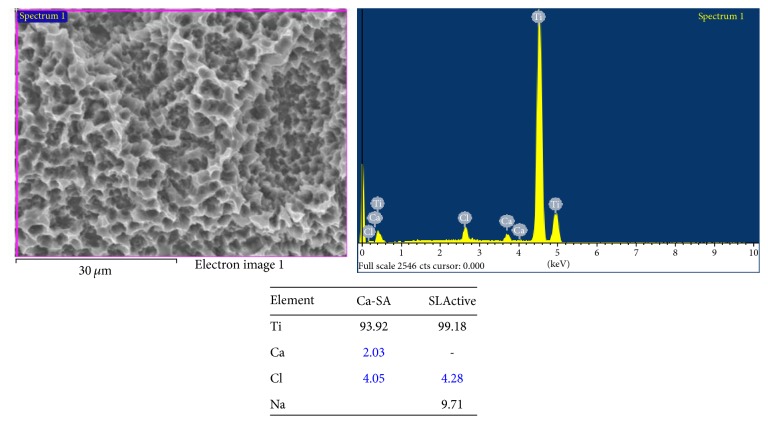
Electron spectroscopy analysis of CA and SLActive surfaces. These surfaces presented comparable percentages of titanium.

The ions distribution on the surface was carbon (C) 2.51%, sodium (Na) 0%, chlorine (Cl) 4.05%, and titanium (Ti) 93.92% (Figure 2)

#### 3.1.2. SLActive Surface

Ten micrographs of this surface were examined. The SLActive roughness (Sa) was 2.72 ± 0.28 *µ*m with a hierarchical structure characterized by irregularly rounded shape domains, incorporating more rounded grooves with sharp-edged and overhanging craterlike micropores (Figure 1). The smooth and amorphous structure of the submicron topography observed at high magnifications is compatible with the oxide film formed during the acidic treatment after sandblasting.

The spectroscopy analysis allowed an evaluation of the elements presented on the implant surfaces. The distribution of carbon was 2.17%, calcium, 0%; sodium, 9.71%; chlorine, 4.21%; and titanium, 99.18% (Figure 2).

### 3.2. Histological Evaluation

Microscopically, all 36 implants were well integrated into bone. Implants were in contact with cortical bone along the upper threads, while the lower threads were in contact with either newly formed bone or marrow spaces. Fibrous tissue was absent between bone and implant surfaces in all the implants of the 2 groups.

### 3.3. 15 Days

#### 3.3.1. CA Surface

**Figure 3:**
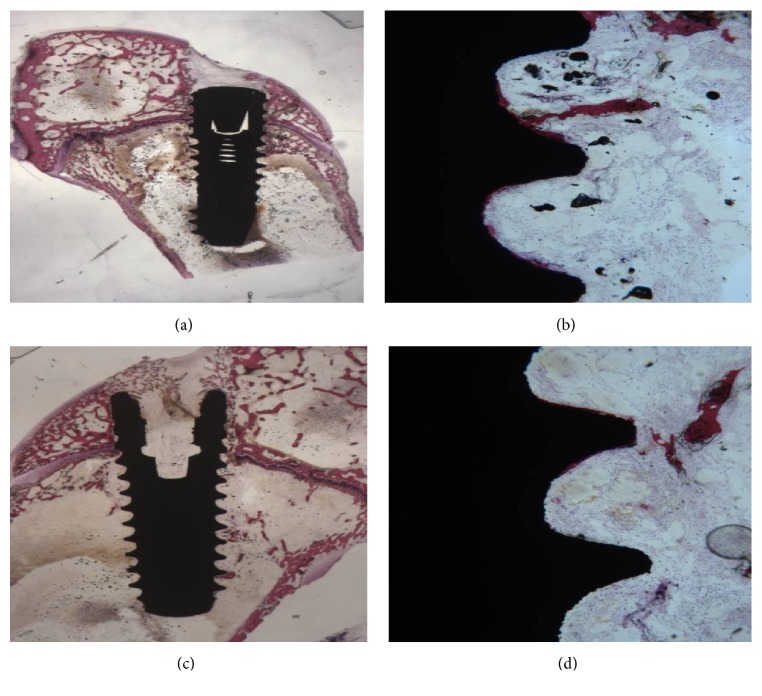
(a) 15-day CA surface. A few newly formed bone trabeculae were present around the implants. Toluidine blue and acid fuchsin 2x. (b) Higher magnification. A few bone trabeculae were present in the implant concavities. Toluidine blue and acid fuchsin 50x. (d) SLActive surface. A newly formed bone was present. Toluidine blue and acid fuchsin 2x. (c) Trabecular bone was found in the concavities. Toluidine blue and acid fuchsin 50x.

Bone trabeculae could be seen in contact with the implant surface; numerous osteoblasts secreting osteoid matrix directly on the implant surface could be observed (Figure 3(a)). A high number of bone trabeculae adjacent to the implants was observed. Small newly formed bone trabeculae, heavily stained with acid fuchsin, were present in the concavities of the threads (Figure 3(b)). A few inflammatory cells were present. A few osteoclasts were observed on the implant surface.

The mean BIC percentage was 21.67 ± 4%.

#### 3.3.2. SLActive Surface

Bone was observed on the implant surface. Many thin bone trabeculae were present in the thread concavities (Figure 3(c)). Only a few inflammatory cells were present. No multinucleated giant cells were present (Figure 3(d)). No mature mineralized bone was observed in the cortical region, while in the marrow spaces many osteoblasts secreting osteoid matrix were present. The mean BIC percentage was 18.5 ± 5.2%.

### 3.4. 30 Days

#### 3.4.1. CA Surface

**Figure 4:**
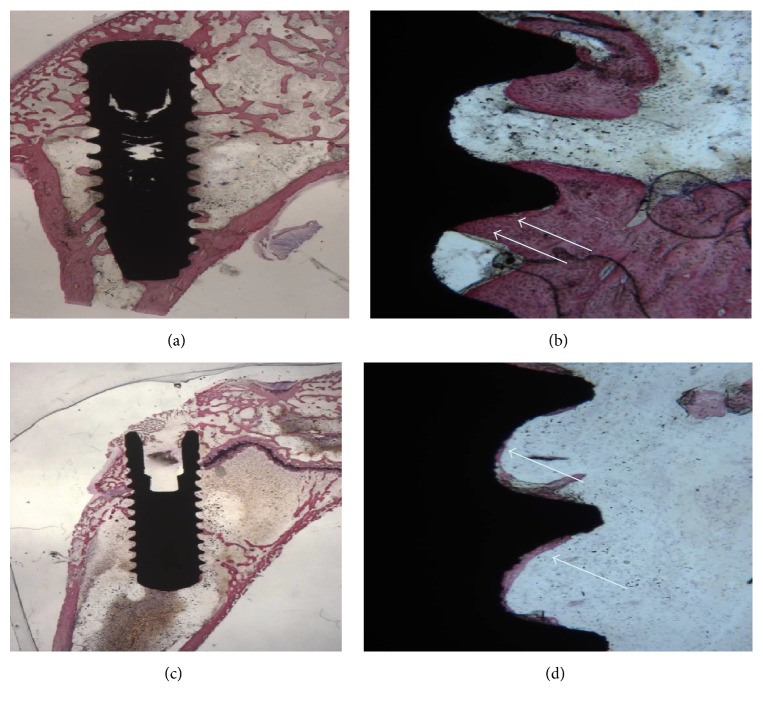
(a) 30-day CA surface. Mature bone was present around the implants. Toluidine blue and acid fuchsin 2x. (b) At higher magnification, more mature bone trabeculae were present around the implant surface (Arrows). Toluidine blue and acid fuchsin 50x. (d) SLActive surface. Histological pictures showing the mature bone in the implant concavities (Arrows). Toluidine blue and acid fuchsin 2x. (c) No gaps are present in the implant bone. Toluidine blue and acid fuchsin 5x.

Mature bone was observed in direct contact with the implant surface; few osteoblasts secreting osteoid matrix were observed on the implant surface. A higher number of bone trabeculae were observed adjacent to the implants, in comparison with the results observed at 15 days (Figures 4(a) and 4(b)). Some osteoclasts were present in the bone around the implants. No multinucleated or inflammatory cells were observed. The mean BIC percentage was 54.5 ± 5.6%.

#### 3.4.2. SLActive Surface

Compact bone was present especially in the area where the implant was in contact with cortical bone (Figures 4(c) and 4(d)). Small bone trabeculae tended in many cases to surround almost the whole surface of the implant. A decreased osteoblastic activity was observed. No multinucleated or inflammatory cells were observed.

The mean BIC percentage was 56.83 ± 4.6%.

### 3.5. 60 Days

#### 3.5.1. CA Surface

**Figure 5:**
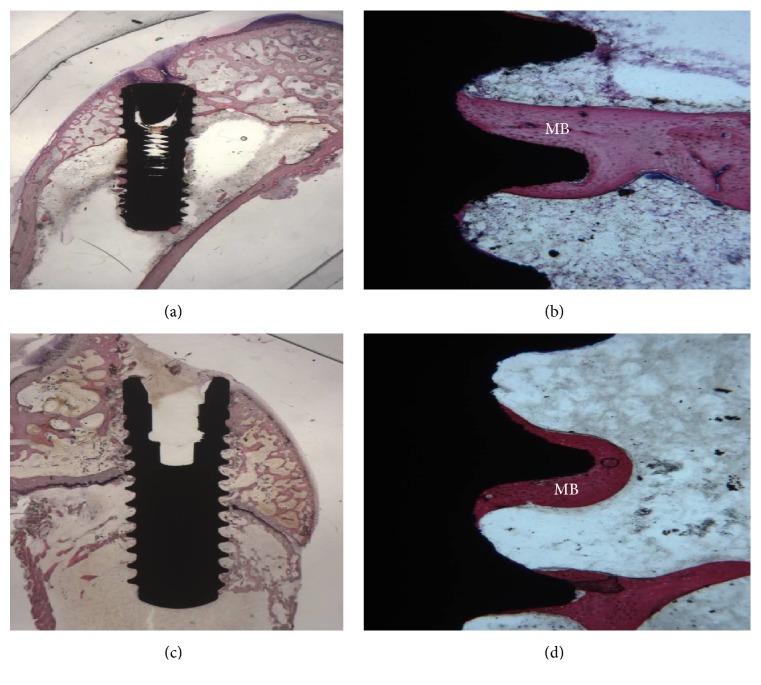
(a) 60-day CA surface. Histological analysis showed a complete bone organization and mineralization. Toluidine blue and acid fuchsin 2x. (b) At higher magnification, mature bone (MB) trabeculae were present in the implant concavities and convexities. Toluidine blue and acid fuchsin 50x. (d) SLActive surface. Mature bone tissue (MB) was observed on the surface of the implants. Toluidine blue and acid fuchsin 2x. (c) No gaps were present at the implant-bone interface. Toluidine blue and acid fuchsin 50x.

At low magnification, mature bone with small marrow spaces could be seen in the cortical portion (Figures 5(a) and 5(b)). Small bone trabeculae were present around the marrow spaces. At higher magnification, a large portion of the implant was lined by newly formed bone. No connective tissue between the bone-implant interface was present. The mean BIC percentage was 62.83 ± 5.8%.

#### 3.5.2. SLActive Surface

No histological differences could be observed compared to the thirty-day observations. Only a few osteoblasts were present. Mature bone was in close contact with the implant surface (Figures 5(c) and 5(d)). Inflammatory cells were present. The mean BIC percentage was 66.6 ± 3.5.

### 3.6. Statistical Evaluation

A little statistical difference was found in the bone-implant contact percentages between the 2 different implant surfaces at 15 days (*p* = 0.027) and at 60 days (*p* = 0.061), while at 30 days (*p* = 0.051) the differences are borderline

## 4. Discussion

It is well established that characteristics of the implants surface, such as nano- and microtopography and physicochemical composition, have a major influence on the outcome of osseointegration, especially at the histological level, aiming at biological and morphological compatibilities [18]. Surface topography and roughness influence the early healing stages of bone integration. Also, surface properties such as wettability, topography, and charge are known to affect endothelial cells attachment and growth, likely by altering the rate of the amount of adsorbed proteins and their conformational change [20].

The main idea behind the establishment of such a rough topography was to increase the surface area of the implant adjacent to the bone and to improve the cell adhesion to the surface, thereby achieving higher bone-to-implant contact and better biomechanical integrity [17]. Also the chemistry and topography of the dental implant surfaces were demonstrated to be able to influence interactions with all blood components [21]. Literature reports have shown that the acid etching process can employ either a hydrochloric acid/sulfuric acid mixture (HCl/H_2_SO_4_) [22–24] or pickling in 2% hydrofluoric acid/10% nitric acid (HF/HNO_3_) [25]. This treatment changes the chemistry, surface free energy, and hydrophilicity of an implant surface playing a decisive role during the initial interaction with proteins and cells in bone. In addition to increasing surface roughness, surface blasting and acid etching could remove surface contaminants and increase the surface reactivity of the metal. The osteoblastic cells play a critical role in the early stages of osseointegration. In the present study, histomorphometry was used to measure the percentage of bone-to-implant contact (BIC) to evaluate osseointegration and the rate of healing of the CA and SLActive surfaces. In fact, BIC is the most important method to determine the percentage of mineralized tissues at the interface of implant and bone [26]. The values of BIC found in the present study were similar to those reported by other authors in vivo studies conducted on animal models [27, 28]. Similar high levels of BIC were found along both surfaces.

Osteoblasts were observed on the implant surfaces during the first healing phase in all histological sections. At 15 days, the osteoblasts produced osteoid matrix directly on the CA and SLActive implant surfaces. In the present study, it was found that the bone formation started preferentially in the implant thread concavities during the early healing periods. This result confirmed also the influence of the implant macrostructure in first phase of healings reported in previous studies in rabbits [29, 30].

At 60 days, both surfaces demonstrated similar healing pattern and bone remodeling, with dense and mature bone deposited upon almost the entire implant surface. A little significant difference in terms of BIC values was observed between implants.

Therefore, the use of implants with different shape and macrodesign had not influenced the bone response. It has been demonstrated that topography may modulate the osteoblast differentiation [31, 32], and rougher surfaces produced increased degree of bone formation around the implants, but, on the other hand, in this study no influence of the macrostructure was observed.

The histological findings showed that both surfaces presented a similar BIC and appeared to be highly osteoconductive.

## 5. Conclusions

Within the limitations of the rabbit model in this study, the bone-implant contact evaluations indicated that a good osteoconduction along the CA and SLActive surfaces occurred during the initial 15 days after implant placement, and a high BIC was reached for both surfaces after 60 days. These results suggested that both implant surfaces could be clinically advantageous for shortening the implant healing period, providing an earlier fixation, and minimizing micromotion, thus allowing earlier loading protocols and restoration of function for implants placed in areas with low density bone.
